# Monitoring the Spatial Spread of COVID-19 and Effectiveness of Control Measures Through Human Movement Data: Proposal for a Predictive Model Using Big Data Analytics

**DOI:** 10.2196/24432

**Published:** 2020-12-18

**Authors:** Zhenlong Li, Xiaoming Li, Dwayne Porter, Jiajia Zhang, Yuqin Jiang, Bankole Olatosi, Sharon Weissman

**Affiliations:** 1 Geoinformation and Big Data Research Laboratory Department of Geography University of South Carolina Columbia, SC United States; 2 Department of Health Promotion, Education, and Behavior Arnold School of Public Health University of South Carolina Columbia, SC United States; 3 Department of Environmental Health Sciences Arnold School of Public Health University of South Carolina Columbia, SC United States; 4 Department of Epidemiology and Biostatistics Arnold School of Public Health University of South Carolina Columbia, SC United States; 5 Department of Health Services Policy and Management Arnold School of Public Health University of South Carolina Columbia, SC United States; 6 Department of Internal Medicine School of Medicine University of South Carolina Columbia, SC United States

**Keywords:** big data, human movement, spatial computing, COVID-19, artificial intelligence

## Abstract

**Background:**

Human movement is one of the forces that drive the spatial spread of infectious diseases. To date, reducing and tracking human movement during the COVID-19 pandemic has proven effective in limiting the spread of the virus. Existing methods for monitoring and modeling the spatial spread of infectious diseases rely on various data sources as proxies of human movement, such as airline travel data, mobile phone data, and banknote tracking. However, intrinsic limitations of these data sources prevent us from systematic monitoring and analyses of human movement on different spatial scales (from local to global).

**Objective:**

Big data from social media such as geotagged tweets have been widely used in human mobility studies, yet more research is needed to validate the capabilities and limitations of using such data for studying human movement at different geographic scales (eg, from local to global) in the context of global infectious disease transmission. This study aims to develop a novel data-driven public health approach using big data from Twitter coupled with other human mobility data sources and artificial intelligence to monitor and analyze human movement at different spatial scales (from global to regional to local).

**Methods:**

We will first develop a database with optimized spatiotemporal indexing to store and manage the multisource data sets collected in this project. This database will be connected to our in-house Hadoop computing cluster for efficient big data computing and analytics. We will then develop innovative data models, predictive models, and computing algorithms to effectively extract and analyze human movement patterns using geotagged big data from Twitter and other human mobility data sources, with the goal of enhancing situational awareness and risk prediction in public health emergency response and disease surveillance systems.

**Results:**

This project was funded as of May 2020. We have started the data collection, processing, and analysis for the project.

**Conclusions:**

Research findings can help government officials, public health managers, emergency responders, and researchers answer critical questions during the pandemic regarding the current and future infectious risk of a state, county, or community and the effectiveness of social/physical distancing practices in curtailing the spread of the virus.

**International Registered Report Identifier (IRRID):**

DERR1-10.2196/24432

## Introduction

COVID-19, which is caused by SARS-CoV-2, was originally detected in Wuhan, China, in December 2019. On March 11, 2020, the World Health Organization (WHO) declared the COVID-19 outbreak a pandemic due to its rapid spread to several geographic regions [1]. To limit the spread of COVID-19, unprecedented measures, such as mass quarantines of cities (eg, Wuhan, China) and lockdowns of entire countries (eg, Italy), have been taken. Due to the rapid human-to-human transmission of COVID-19, models or measurements that contribute to increased knowledge about potential infectious risk at different geographic levels can play an essential role for residents, medical workers, and governments. Such models can help local authorities and communities better allocate resources and efforts at a community level. Meanwhile, it is equally important for policy makers and emergency responders to understand how people practice social/physical distancing and how effective these control measures are at curbing the spatial propagation of the virus.

Human movement is an important driver of the geographic spread of infectious diseases [2]. For example, studies on severe acute respiratory syndrome (SARS) [3], Middle East respiratory syndrome (MERS) [4], and influenza H1N1 [5,6] all confirmed that airline travel was a major contributor to virus transmission on a large spatial scale. From a public health perspective, prediction and control of the spread of infectious diseases benefits greatly from our growing capacity to quantify human movement [7]. COVID-19 has a high human-to-human transmission rate and can be transmitted during the preclinical incubation period. So far, limiting and tracking human movement during the outbreak has proven effective at reducing the spread of COVID-19 in different countries [8-10]. In this sense, monitoring and analyzing human movement patterns or population flows at different spatial scales (global, country, state, county, and community) is critical for us to gain a better understanding of the current and future infectious risk at a population level during the pandemic. Such situational awareness can help governments at all levels (local, state, federal, and international) proactively reallocate medical supplies and medical workforces to more vulnerable areas, enabling better preparation and readiness for disease outbreaks.

Existing studies have used various data sources to quantify human movement and model the spread of infectious diseases. On a large scale, airline data are important sources in understanding global transmission of infectious diseases. For example, global spread of SARS simulation models have been generated with airline data [11]. Although airline data deepened our understanding of the transmission mechanism of infectious diseases at large geographical scales, the data have shown a limited usefulness for understanding transmission across short distances [12,13]. On a local or regional scale, mobile phone data have been used as a measurement of human mobility; such data improved our understanding of spatial transmission patterns of malaria [14], cholera [15], and influenza [16]. Due to privacy issues, mobile phone data are generally limited in terms of accessibility and are often limited to a local region or one country; therefore, this data cannot provide systematic global coverage [17]. Besides mobile phone data, commuting patterns derived from census data also play an important role in understanding virus spread patterns on a local scale [13,18].

With the increasing prevalence of location-enabled social media, geotagged Twitter data have been widely used in human mobility studies (eg, [19-21]), yet limited research has been conducted to validate the potential and limitations of these data for studying human movement at different geographic scales (eg, from global to local) in the context of global infectious disease transmission. Meanwhile, the recent development of artificial intelligence (AI) has proven useful for diagnosis, drug analysis, data collection, and outbreak prediction [22]. Various types of neural network algorithms have demonstrated capacity in predicting HIV epidemics [23], influenza-like illness [24], and SARS [25]. However, the majority of these AI-based prediction algorithms have focused on mathematical models of trend development and outbreak identification, in which limited geospatial information (especially at different geographic scales) is considered. The recent COVID-19 pandemic provides us with a unique opportunity to explore innovative approaches to effectively use big data from Twitter and AI-based algorithms, and examine their efficiency in enhancing situational awareness and risk prediction in public health emergency response and disease surveillance systems.

By leveraging the interdisciplinary team’s collective expertise in spatiotemporal modeling, big data analytics, infectious disease, spatial epidemiology, and health promotion and behavior modification, we propose to develop a novel data-driven public health approach using big data from Twitter coupled with other human mobility data sources and AI to monitor and analyze human movement at different spatial scales (from global to regional to local). With the proposed approach, we aim to answer the following critical questions relating to the COVID-19 pandemic:

1. Where are people coming from and going to during the pandemic? We will answer this question by developing an Origin-Destination-Time data cube (ODT cube) to efficiently extract historical and near real-time population flows from worldwide geotagged tweets.
2. What is the current and future infectious risk of a country, state, or county? This will be estimated using a spatial-temporal fused neural network considering historical human movement patterns and real-time population flows.
3. How well are people following the social/physical distancing orders? This question will be examined by performing spatial-temporal aggregation of the ODT cube at different spatial scales and temporal resolutions to quantify human movement at different spatial scales.
4. How effective is social/physical distancing for curtailing the spread of the virus? We will answer this question by conducting spatiotemporal and geostatistical analysis (eg, regression and correlation) for the aggregated population flows, the daily confirmed cases, and other factors such as face mask policies.

The answers to these questions will be compiled as maps, diagrams, news releases, technique reports, and peer-reviewed journal articles.

## Methods

### Data Collection and Database

This project will collect the following 4 types of data worldwide (where data are available): (1) geotagged Twitter data, (2) daily confirmed COVID-19 cases at the available highest spatial resolution for all countries, (3) the most recent socioeconomic and demographic information (at the county level in the United States and a similar level of administrative unit for other countries), and (4) human movement information from other mobility data sources, such as mobile phone–based mobility data (eg, SafeGraph [26] and Descartes Labs [27]), the Google Mobility report [28], and the Apple Mobility report [29]. We have developed a computer program to stream geotagged tweets using Twitter’s Standard (free) streaming application programming interface (API). In addition, we will subscribe to Twitter’s Decahose API for a limited time period, which delivers a 10% random sample of real-time full Twitter streams [30]. Worldwide historical geotagged Twitter data collected by the team over the past 5 years will be used to construct past population flows and identify spatiotemporal patterns of human movement. Building upon our previous work on indexing and processing geospatial big data [31,32], we will develop a scalable database to store and manage the aforementioned multisource data sets. The database will be indexed with multilevel spatial scales (eg, country, state, and county) and temporal resolutions (eg, year, month, day, and hour) and will be connected to our in-house Hadoop computing cluster for efficient big data computing, analytics, and visualizations.

### Analytic Approach

#### Develop an ODT Data Cube for Efficient Analysis of Human Movement From Geotagged Tweets With Varying Spatiotemporal Scales

**Figure 1:**
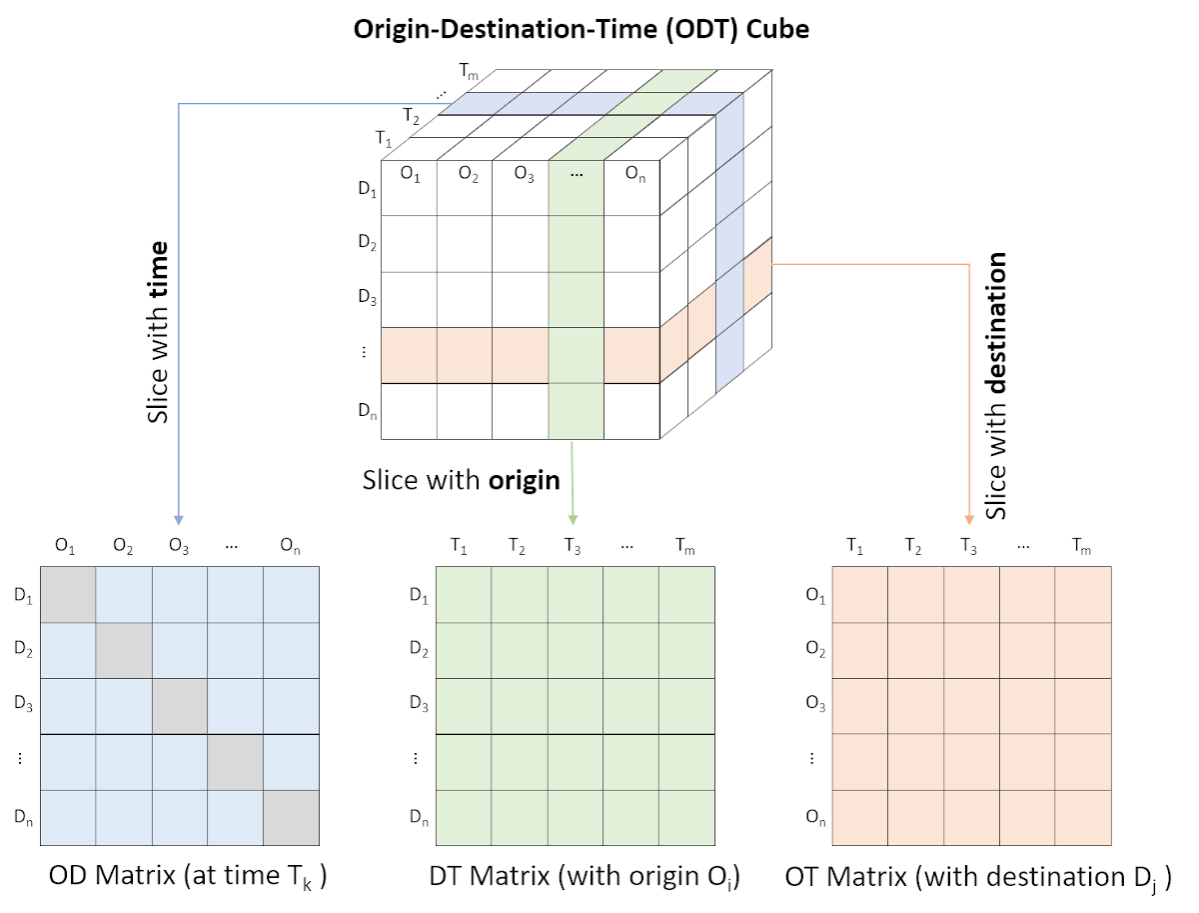
Illustration of Origin-Destination-Time data cube for modeling human movement.

Data cube has been widely used to model high-dimensional spatiotemporal data (eg, [33,34]). We will develop an ODT data cube as a high-level conceptual model for quantifying human movement across different places or locations over time (Figure 1) from billions of geotagged tweets. The ODT cube will serve as a foundation data model for efficiently conducting human movement analysis at different spatial and temporal scales. In the ODT cube, origin (O) and destination (D) are a set of places or locations (eg, administrative boundaries such as county, state, and country, or latitude/longitude grids) that can be displayed on a map. Each cell in the data cube has a value that indicates the number of people that moved from the origin location to the destination location during a specific time period (eg, an hour, day, or month). In other words, each cell value indicates the connection (measured by population movement) between two locations. Using the ODT cube, we can efficiently retrieve the number of people that moved from *O_i_* to *D_j_* at time *T_k_*.

In total, 3 types of matrices will be derived from the data cube: the origin-destination (OD) matrix quantifies the population flows between all the origin and destination locations during a time period. The destination-time (DT) matrix captures the number of incoming people to all destination locations from a specific origin location over a series of times, while the origin-time (OT) matrix captures the number of outgoing people from all origins to a specific destination over a series of times. In addition, the number of unique Twitter users can be calculated for a specific location over time. This enables us to efficiently conduct spatial-temporal aggregations of human movement at varying spatial and temporal resolutions.

The OD matrix is an *n* ´ *n* matrix, where *n* is the number of geographic entities included in the study. Column *O_x_* and row *D_x_* are the same location (x). An entry *v_ij_* in this matrix represents the number of people moving from origin *i* to destination *j*. It should be noted that human movements are directional. Therefore, *v_ij_* and *v_ji_* stand for two different spatiotemporal movements that are likely to have different values. We define the values in the diagonal cells (grey cells in the OD matrix), *v_ii_,*as the number of unique Twitter users in location *i*.

The process of constructing the ODT cube is extremely data- and computationally intensive because we need to perform a large number of point-in-polygon spatial operations, and the output will contain billions of connections. We will leverage our expertise in geospatial big data computing to perform the computation using an in-house Hadoop-based computing cluster. Based on the generated ODT cube, we will further derive a number of indices to quantify human mobility at varying spatiotemporal scales including, for example, the daily number of Twitter visitors, daily number of movements (inflow, outflow, intraflow), average travel distance, and place connectedness index between two counties.

#### Develop Population-Level Infectious Risk Maps at Different Spatial Scales Based on Population Flows to Enhance Situational Awareness

The ODT cube quantifies human movement among different places (eg, US counties or census tracts) during a given time period. Knowing such movement information is essential for assessing infectious risk at the population level in a given place. We propose to model the current infection risk of a given place (eg, county) by integrating the following information: (1) population flows derived from the ODT cube during the recent time period among all places (eg, past 14 days), (2) the number of total COVID-19 cases for each place, and (3) socioeconomic and demographic variables that relate to the infection risk of that location (eg, a county’s population density and age and race distributions).

**Figure 2:**
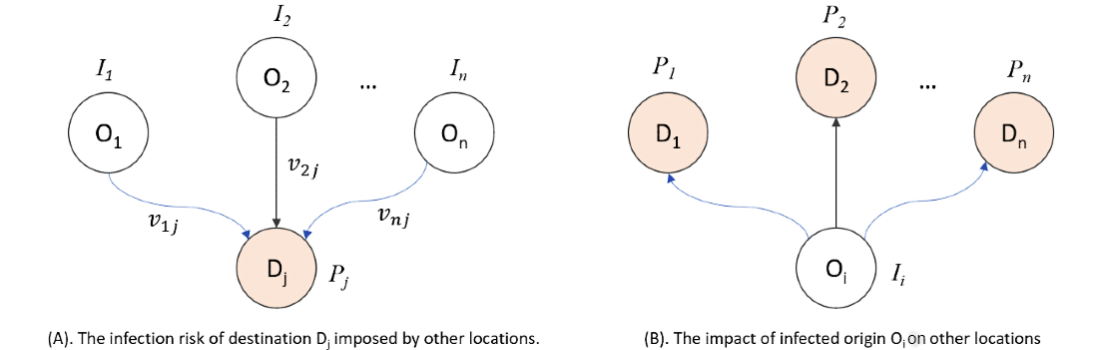
Illustration of (A) infection risk modeling based on the incoming population to a location and (B) the impact modeling of an infected location on other locations.

We will create an infection risk index for each place by combining the abovementioned factors. For example, suppose that, based on the ODT cube, we observe a significant population flow from county *A* to county *B* during the past week and county *A* already has a number of COVID-19 cases, then the infectious risk for county *B* is high (people from a highly infected area are likely to carry the virus). Note that the real scenario is more complex due to the fact that the risk of county *B* is also affected by other counties with confirmed cases that have connections with county *A* and that population movement is not the only factor for infectious risk. In other words, the infection risk of destination *D_j_* can be considered a function of local factors (*P_j_*), combined with population flow from each origin (*v*_1_*_j_*, *v*_2_*_j_*, …*v*_n_*_j_*) weighted by the number of cases at each origin (*I_1_, I_2_, ..., I_n_*; Figure 2A). A risk index will be calculated for each location to produce an infectious risk map. Based on the ODT data cube, risk map generation can be efficiently implemented using matrix computation. Such risk maps would be useful for targeting surveillance and outbreak control activities for a region.

Besides modeling the infection risk of a location using the incoming populations, we will also estimate the risk impact of a location with confirmed cases on other locations. For example, since Italy was severely infected at the early stage of the pandemic, it would be helpful to understand where the outgoing population from Italy traveled to. As illustrated in Figure 2B, we will build a model that combines the population movement information between the targeted location (*O_i_*) and other locations (*D_1_, D_2_, ..., D_n_*), as well as other factors associated with each location (*P_1_, P_2_, ..., P_n_*). The output of the model will be a map showing the potential impact of the incoming populations from the targeted location (eg, Italy).

#### Develop a Predictive Model to Estimate Future Infectious Risk Using a Fused Neural Network by Considering Both Spatial Patterns and Temporal Trends of the Population Movement

In this research task, we aim to explore the feasibility and performance of a predictive model for future infectious disease potential at the US county level based on the following information: (1) near real-time human movement information (from real-time Twitter data streams), (2) the daily case count of each county (will be collected/compiled each day), and (3) other factors such as socioeconomic and demographic information.

**Figure 3:**
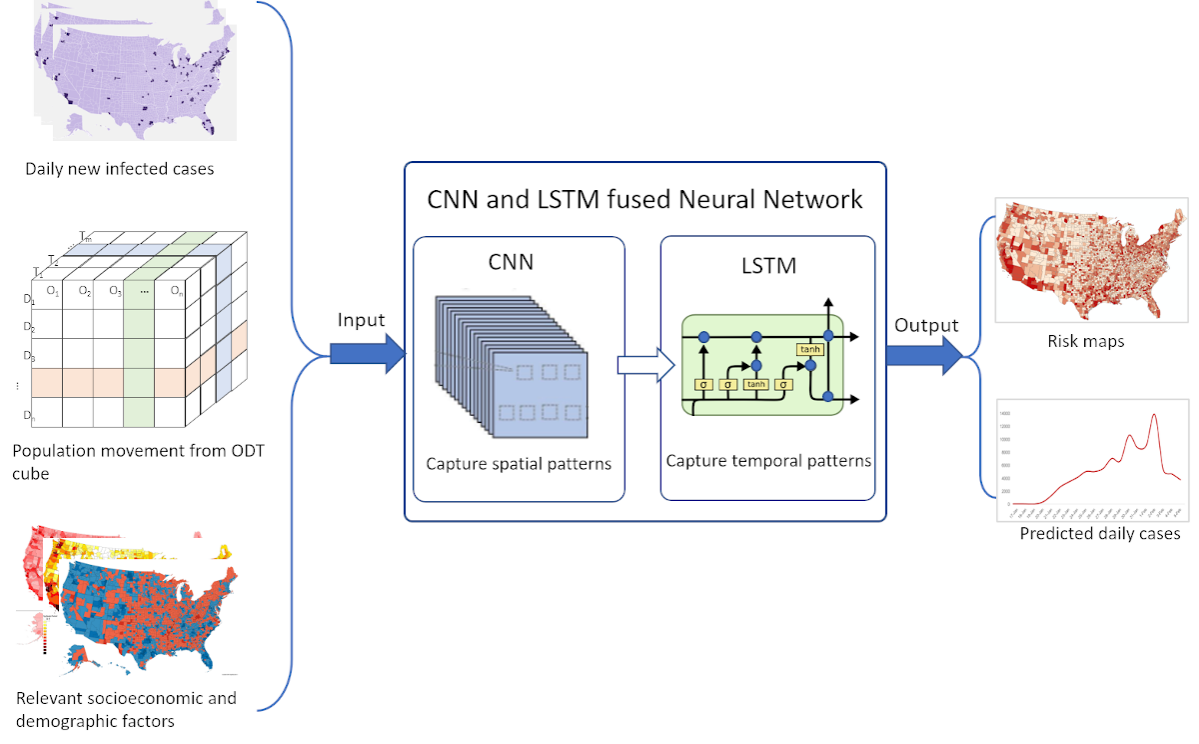
Conceptual architecture of the CNN-LSTM fused neural network for infectious risk prediction. CNN: convolutional neural network; LSTM: long short-term memory recurrent neural network; ODT: Origin-Destination-Time.

Given the complex epidemiological and geographic processes of different infectious factors, we propose to use deep learning to explore complex infectious processes using the large volumes and high dimensions of the input data. Deep learning is one type of machine learning in AI. Unlike traditional machine learning, in which the parameters of an algorithm (eg, support vector machine) are configured by experts, deep learning determines these parameters by learning the patterns in a large amount of data based on artificial neural networks. Specifically, we will develop a fused neural network that integrates two types of neural networks, convolutional neural network (CNN) and long short-term memory recurrent neural network (LSTM), to consider spatial patterns and temporal trends simultaneously in the predictive model (Figure 3). The fused neural network will include a series of CNN layers in the front end followed by LSTM layers with a Dense layer on the output. The locations in the ODT cube (eg, counties) would be treated as pixels (neurons) in the CNN network to capture spatial relationships and local patterns, and the temporal trend will be predicted with the LSTM network. Different combinations of socioeconomic and demographic factors will be tested during the model building, training, and validation process, and the combination yielding the highest accuracy will be used in the final model.

### Ethics and Dissemination

This research does not involve human subjects and received an exempt review from the Institutional Review Board (IRB). All data collected in this project are in the public domain. Twitter data are collected using the official Twitter API. We are fully aware of the potential privacy concerns related to handling geotagged tweets, which contain location information and may include some personal information provided by the users directly. We have been following and will continue to follow Twitter developer policies strictly when collecting and sharing Twitter data. The raw individual tweets with exact latitude and longitude will not be published in any format, including maps, technical reports, or journal publications. All data collected in this study will be stored in an in-house Hadoop computing cluster hosted in a secure server room at the University of South Carolina with firewall protection, two-factor authentication, and endpoint security. The results of this project will be disseminated as maps, summary graphics, news reports, research articles, and interactive web portals.

## Results

This project was funded as of May 2020. We have started the data collection, processing, and analysis, and have built a spatial web portal for sharing the human mobility data extracted from geotagged tweets and SafeGraph data [35].

## Discussion

### Overview

In this paper, we report a research protocol that will use big data from social media to derive information on human movement or population flows to monitor the spatial spread of COVID-19, quantify the effectiveness of control measures, and predict the current and future infectious risk at various geospatial scales. We believe geotagged Twitter data are sufficient for studying population flows on a large spatial scale with low or medium spatial resolutions, such as the movement between countries and between states in the United States. For the county level, our previous studies indicate that these data perform well for examining human movement between different US counties [36-38]. For finer resolutions than county, we have successfully conducted human mobility studies at the census tract level [21] and street/community level within a city [39]. However, we are aware that studies at a spatial resolution higher than city or county only work in highly populated areas since at this resolution we can only use tweets with exact coordinates. Considering this issue, we will only perform community-level analysis for highly populated cities (eg, New York City) when using Twitter-derived population flows.

Another limitation we would like to point out is that Twitter data has intrinsic demographic and socioeconomic biases as suggested in a few studies [40-42]. Despite this limitation, Hawelka et al [19] confirmed that geotagged tweets are exceptionally useful for quantifying country-to-country population movement. Our recent study also suggests that the county-level population movement derived from Twitter data can accurately reflect regular (eg, holidays) and nonregular (eg, hurricanes) events [36]. The third issue is that Twitter users’ tweeting behavior and Twitter’s APIs and platform change over time and may continue to change in the future, which affects the volume of streamed geotagged tweets. For example, Twitter removed support for precise geotagging in June 2019 [43] and Twitter users may stop geotagging their posts due to privacy concerns. To tackle the aforementioned limitations of geotagged tweets, we will integrate human mobility data derived from other aforementioned data sources including SafeGraph (which provides US Census Block Group–level human movement information) to better capture and quantify human movement during the pandemic [44].

### Conclusions

Human movement is among the essential forces that drive the spatial spread of COVID-19. During a global pandemic, monitoring and analyzing human movement patterns or population flows is critical for us to gain a better understanding of current and future infectious risk at the population level. This research aims to use big data from a social media site (Twitter), AI, and spatiotemporal analysis to monitor and model the spatial spread of COVID-19 at different spatial scales (from local to regional to global) through the lens of human movement. The results of this study will not only provide enhanced situation awareness for the government at all levels, but also offer valuable contributions for building collective public awareness of the role people play in the evolution of the COVID-19 crisis.

The findings of this research may also have implications for policy by assisting the policy makers and general public to evaluate the effectiveness of various control measures that aim to reduce human movement during the pandemic. For example, the debate about the true effectiveness of social distancing as a public health tool for limiting COVID-19 transmission requires mobility research to generate evidence-based guidance [45]. This is especially important in the context of mixed research findings about COVID-19 aerosolization [40,46,47] and the true effectiveness and costs of social distancing [48,49]. As universities and schools reopen, and traditional socialization activities like sporting and musical events resume, measuring and tracking the impact of human mobility will take on greater significance.

We hope that the results can help government officials, public health managers, emergency responders, and researchers to answer critical questions during the pandemic as elaborated above. Although this research is a response to the current COVID-19 pandemic, the proposed research will make significant contributions to data sources, applications, models, and methodology for a variety of human mobility studies. This research is expected to have a broad impact on diverse fields that can benefit from a better understanding of human movement at varying spatial scales, such as infectious disease spread in public health, transportation, tourism, and economics.

## Abbreviations

AI: artificial intelligence
API: application programming interface
CNN: convolutional neural network
DT: Destination-Time
MERS: Middle East respiratory syndrome
LSTM: long short-term memory recurrent neural network
ODT: Origin-Destination-Time
OD: Origin-Destination
OT: Origin-Time
SARS: severe acute respiratory syndrome
WHO: World Health Organization
